# Supplementary material for: Metabolic response to an acute bout of mild dynamic exercise performed under normobaric moderate hypoxia: A NMR-based metabolomics study
Source: PLoS One. 2025 Jul 1;20(7):e0325447. doi: 10.1371/journal.pone.0325447 (PMC12212504; doi:10.1371/journal.pone.0325447)
Supplement: S1 Fig — Heatmap illustrating the matrix of Pearson correlation coefficients of significantly altered plasma (A) and urine (B) metabolites after the HYPO test: blu, positive correlations; red, negative correlations. * p < 0.05; ** p < 0.01; *** p < 0.001; **** p < 0.0001. (PDF) [file pone.0325447.s001.pdf]

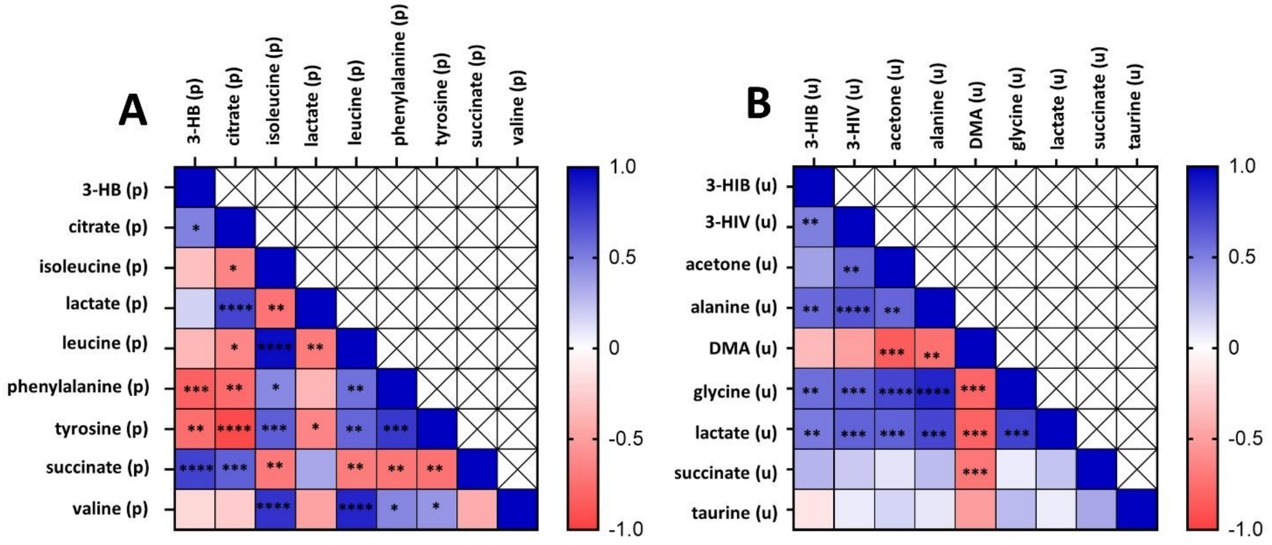

S1 Fig. Correlation analysis. Heatmap illustrating the matrix of Pearson correlation coefficients of significantly altered plasma (A) and urine (B) metabolites after the HYPO test: blu, positive correlations; red, negative correlations. * $p < 0.05$; ** $p < 0.01$; *** $p < 0.001$; **** $p < 0.0001$.
