# Supplementary material for: Metabolic response to an acute bout of mild dynamic exercise performed under normobaric moderate hypoxia: A NMR-based metabolomics study
Source: PLoS One. 2025 Jul 1;20(7):e0325447. doi: 10.1371/journal.pone.0325447 (PMC12212504; doi:10.1371/journal.pone.0325447)
Supplement: S2 Fig — The figure illustrates the estimated statistical power as a function of total sample size, based on a post-hoc analysis conducted using G*Power. The analysis refers to the main effects of exercise, oxygen exposure, and their interaction. Effect sizes (η²) were computed as the average of those obtained for the metabolites that showed statistically significant differences in the repeated measures ANOVA. (PDF) [file pone.0325447.s002.pdf]

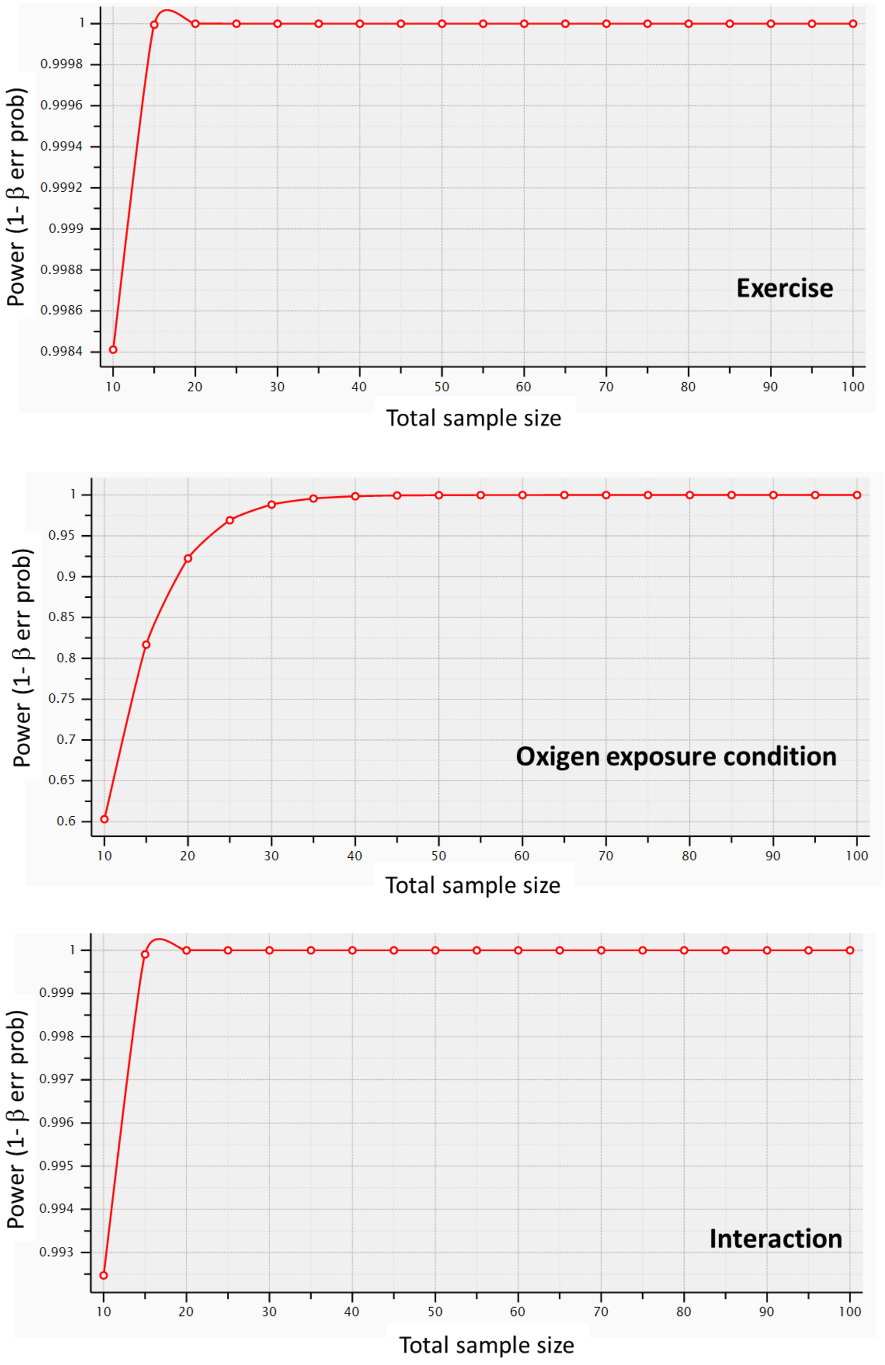

S2 Fig. Post-hoc power analysis related to repeated measures ANOVA on plasma metabolites. The figure illustrates the estimated statistical power as a function of total sample size, based on a post-hoc analysis conducted using G*Power. The analysis refers to the main effects of exercise, oxygen exposure, and their interaction. Effect sizes (η^2) were computed as the average of those obtained for the metabolites that showed statistically significant differences in the repeated measures ANOVA.
