# Supplementary material for: Metabolic response to an acute bout of mild dynamic exercise performed under normobaric moderate hypoxia: A NMR-based metabolomics study
Source: PLoS One. 2025 Jul 1;20(7):e0325447. doi: 10.1371/journal.pone.0325447 (PMC12212504; doi:10.1371/journal.pone.0325447)
Supplement: S1 Table — Factors: oxygen exposure condition and exercise. Statistical significance was set at p-value < 0.05. Partial eta squared (η²) values indicate effect sizes. (PDF) [file pone.0325447.s003.pdf]

S1 Table. Two-way ANOVA analysis of plasma metabolite levels. Factors: oxygen exposure condition and exercise. Statistical significance was set at p-value < 0.05. Partial eta squared (η^2) values indicate effect sizes.

	p-value **(η^2)**		
**Metabolites**	**Exercise**	**Oxygen exposure condition**	**Interaction**
2-hydroxyisovalerate	0.995 (0)	0.778 (0.007)	0.275 (0.098)
2-hydroxybutyrate	0.066 (0.254)	0.045 (0.293)	0.249 (0.109)
3-hydroxyisobutyrate	0.933 (0.001)	0.209 (0.128)	0.149 (0.165)
3-hydroxybutyrate	0.073 (0.243)	0.019 (0.378)	0.001 (0.602)
Acetone	0.66 (0.017)	0.858 (0.003)	0.171 (0.150)
Alanine	0.22 (0.122)	0.359 (0.071)	0.675 (0.015)
Carnitine	0.278 (0.097)	0.017 (0.392)	0.02 (0.374)
Choline	0.096 (0.214)	0.511 (0.037)	0.391 (0.062)
Citrate	0.024 (0.326)	0.147 (0.167)	0.054 (0.274)
Creatinine	0.196 (0.135)	0.624 (0.021)	0.160 (0.158)
Dimethylamine	0.643 (0.018)	0.696 (0.013)	0.357 (0.071)
Dimethylglycine	0.306 (0.087)	0.341 (0.075)	0.202 (0.131)
Glycerol	0.821 (0.004)	0.655 (0.017)	0.658 (0.017)
Glycine	0.069 (0.250)	0.804 (0.005)	0.766 (0.008)
Glucose	0.541 (0.031)	0.049 (0.285)	0.014 (0.410)
Glutamine	0.576 (0.027)	0.056 (0.271)	0.644 (0.018)
Histidine	0.461 (0.046)	0.305 (0.087)	0.653 (0.017)
Isobutyrate	0.565 (0.028)	0.325 (0.081)	0.762 (0.008)
Isoleucine	0.001 (0.765)	0.399 (0.060)	0.014 (0.407)
Lactate	0.106 (0.678)	0.284 (0.095)	< 0.001 (0.677)
Leucine	< 0.001 (0.592)	0.746 (0.009)	0.027 (0.346)
Lysine	0.992 (0)	0.405 (0.059)	0.176 (0.147)
Methanol	0.817 (0.005)	0.388 (0.062)	0.935 (0.001)
Methionine	0.595 (0.024)	0.490 (0.039)	0.326 (0.080)
Ornithine	0.198 (0.134)	0.358 (0.07)	0.416 (0.056)
Phenylalanine	0.033 (0.325)	0.789 (0.007)	0.251 (0.110)
Proline	0.08 (0.234)	0.283 (0.095)	0.578 (0.026)
Pyruvate	0.508 (0.037)	0.752 (0.009)	0.002 (0.578)
Succinate	0.003 (0.542)	0.069 (0.250)	0.002 (0.570)
Taurine	0.441 (0.050)	0.307 (0.087)	0.381 (0.065)
Tyrosine	0.01 (0.436)	0.875 (0.002)	0.125 (0.185)
Urea	0.997 (0)	0.573 (0.027)	0.306 (0.087)
Valine	0.008 (0.455)	0.522 (0.035)	0.023 (0.362)
