# Supplementary material for: Metabolic response to an acute bout of mild dynamic exercise performed under normobaric moderate hypoxia: A NMR-based metabolomics study
Source: PLoS One. 2025 Jul 1;20(7):e0325447. doi: 10.1371/journal.pone.0325447 (PMC12212504; doi:10.1371/journal.pone.0325447)
Supplement: Attachment — Submitted filename: Response to Reviewers.pdf [file pone.0325447.s006.pdf]

UNIVERSITÀ DEGLI STUDI
DI CAGLIARI

Flaminia Cesare Marincola
Dipartimento di Scienze chimiche e geologiche
Cittadella universitaria di Monserrato
SS 554
09042, Monserrato, Cagliari
ITALY

Reviewer #1: Prof. Krzysztof Kusy

This is an interesting metabolomic study on the effect of short-time (3-min) low-intensity exercise. The metabolic response (plasma and urine) was assessed using robust up-to-date methods, which is the study's strongest point. The measurement procedures seem to be planned and executed properly. I fully appreciate the work done and the meticulous analysis. However, there is a serious problem with terminology, resulting in huge over- and misinterpretation of the obtained data, partly inadequate discussion, and exaggerated unjustified conclusions. The Authors, throughout the manuscript, continually confuse "(competitive) sport" with "leisure time physical/sporting activity", "athletes" with "non-competitive leisure exercisers", and, finally "training" with "exercise". Below I try to explain key terms.

General comments

Comment: The definition for competitive athlete as it is suggested to be used in, but not limited to, health and medical science research is available here: https://www.acc.org/latest-in-cardiology/articles/2016/06/27/07/06/the-terms-athlete-and-exercisers or here: *Scand J Med Sci Sports. 2016 Jan;26(1):4-7. doi: 10.1111/sms.12632.*

Briefly, there are several criteria, which must ALL be fulfilled to define someone as an athlete (performance improvement, participating in sport competition, formally registered in a sport federation, sport/competition as major activity and focus exceeding the time allocated to other professional or leisure activities). In this light, the study participants are not athletes, they probably meet or are above the minimal WHO criteria included in the guidelines on physical activity and sedentary behavior. They just participate in leisure time sporting/physical activity, which refers to all of the behavior connected with physical activity that people engage in their freely disposable time. That they are not competitive athletes may be also concluded from their maximum power achieved at VO2max (255 W; athletes typically exceed 400 W). Also, their VO2max is rather low or moderately above the population average at most (about 30 or 70 percentile of normal population, depending on assignment to age group; see e.g. BMJ Open 2018, 8:e018697, doi: 10.1136/bmjopen-2017-018697; Mayo Clin Proc 2022, 97(2):285-293, https://doi.org/10.1016/j.mayocp.2021.08.020).

Since the research presented here does not involve athletes, the authors should not comment or speculate on it, and in particular cannot call their subjects athletes.

Answer: We thank Prof. Kusy for the insightful comments and the provided references regarding the definition of a competitive athlete. We fully agree with his assessment that the participants in

our study do not meet the criteria to be classified as competitive athletes, as outlined in the mentioned sources. We have therefore revised the manuscript accordingly to ensure clarity and precision in describing our study population. In line with his suggestions, we have removed any references or speculations pertaining to athletes and have adjusted our terminology to accurately reflect the nature of our participants, who engage in leisure-time physical activity rather than competitive sports.

Comment: In addition, the exercise intensity used was 30% of W_{max} attained during the preceding test until exhaustion. In terms of competitive sports (and not only), it is a very low intensity. This is much lower than lactate threshold in athletes (~64-84% of Wmax, depending on the assessment method; see e.g. BMC Sports Sci Med Rehabil 2020, 12, 70, https://doi.org/10.1186/s13102-020-00219-3) and probably even lower than the intensity at lactate threshold in healthy non-athletic adults (was any metabolic threshold determined in this study?). 30% of Wmax in normoxia means the lowest intensity zone used in endurance training, called 'active recovery' (or 'very light'). I would venture the hypothesis that professional athletes, even in hypoxia, would remain in this lowest zone at an intensity of 30% W_{max} and a significant metabolic response would hardly occur. Especially since the duration of this single exercise (3 minutes) at such a low intensity is too short to be an effective exercise stimulus for competitive athletes. As is cited I the introduction, other research teams used exercise under hypoxia lasting several tens of minutes (and of higher intensity).

Answer: Thank you for your insightful comment. We agree that 30% of W_{max} corresponds to a low intensity. To address this point and provide a more comprehensive view of the characteristics of the enrolled subjects, we have integrated additional data into Table 1, which now includes measurements taken at the first ventilatory threshold.

Comment: Anyway, conclusions for competitive sports and training cannot and most not be drawn from the research presented. Even if one assumes that certain metabolic mechanisms are universal, the metabolic adaptations of the competitive athlete (plus inborn predispositions) usually yield a metabolic profile extremely different (quantitatively and qualitatively) from that of the 'average' population as numerous studies show. In addition, there are fluctuations in adaptive metabolic changes during successive phases of the annual training cycle (e.g., general preparation, special preparation, pre-competition/taper phase, competition phase). By the way, had I read only the last sentence of the main text (lines 552-3), I would have erroneously concluded that the subjects were elite athletes being followed in a long training cycle. This is misleading and completely impossible on the basis of this research!
Training is a broad term, however, it always refers to a longer systemic PROCESS of developing skills, knowledge or, as in athletes, mainly (but not only) physical fitness and performance. The concept of exercise, as it is used in this study in a narrow physiological sense, means only a single bout of effort accompanied by resulting metabolic response. It takes at least several weeks or even months of planned and systematically repeated sessions and series of specific exercise bouts constituting the (physical) training to yield adaptive changes. The presented study does not

involve any long-term process and therefore the authors cannot and must not comment on it, in particular interpret the results in the context of sports training.

To sum up, the manuscript should therefore be thoroughly rewritten: **unwarranted references/comments/speculations/conclusions related to competitive sports, athletes and training should be removed, and instead content related to the response to a single low-intensity exercise bout in healthy young adults should be presented.** Below is the list of places were the unwarranted terms were used:

- athlete(s): Lines 27, 49, 53, 62, 91, 113, 261, 276, 406, 413, 415, 482, 510, 529, 539, 543, 553
- sport(s): Lines 58, 71, 73, 107, 259.
- training: Lines 26, 55, 58, 60, 73, 426, 468, 484, 520, 534, 540, 542, 545, 553

Answer: We agree with the recommendations of Prof. Kusy. We have rewritten the manuscript to address his concerns and removed all unwarranted references, comments, speculations, and conclusions related to competitive sports, athletes, and training. Instead, we have focused the content on the response to a single low-intensity exercise bout in healthy non-athletic young adults.

Comment: The most interesting observation to me from this study is that significant metabolic changes can be induced in a healthy young person under hypoxia even with surprisingly low-intensity and short-duration exercise (one that does not induce changes in normoxia). Please try to propose/discuss any application of these results in a healthy non-athletic population. Could it be an aid to recreational training? Replacement of any form/modality of exercise? Pre-adaptation for something? Health prevention? Rehabilitation? In what cases/situations? (instead of speculating on competitive athletes).

Answer: Thanks for highlighting the interesting aspect of our findings. We agree they could open up several potential applications for a healthy non-athletic population.

Following reviewer's suggestions, we have expanded on the broader significance of our results beyond competitive sports. We have now emphasized their relevance to non-competitive individuals, including recreationally active or sedentary people, as well as clinical groups. In the introduction, we have discussed how the exposure to hypoxic environment may benefit these populations by helping manage conditions such as hypertension and obesity, and by supporting rehabilitation efforts through accelerated recovery and improved functional outcomes. Additionally, we have highlighted how it can be used as a pre-adaptation strategy, preparing individuals for low-oxygen environments, such as high-altitude hiking or travel, reducing the risk of altitude sickness. Thus, studying how non-competitive individuals physiologically and metabolically adapt to hypoxic conditions can offer valuable insights for designing more effective rehabilitation and health interventions for these populations.

Other comments

1. Can you please explain/justify why an intensity of 30% Wmax and an exercise duration of 3 min was used?

Answer: A significant body of research in the literature has focused on the effects of prolonged hypoxia on cardiovascular and metabolic parameters of athletes; however, there is still a gap in the literature regarding the impact of very brief exposures to hypoxia. Our laboratory has focused its recent efforts on investigating the effects of acute (a few minutes) normobaric hypoxia on cardiovascular and metabolic parameters of competitive and non-competitive subjects, particularly during low-intensity exercise (1-5) This research aims to fill gaps in understanding how brief hypoxic exposure influences key physiological functions across diverse populations.

References

1. *Hemodynamics and cerebral oxygenation during acute exercise in moderate normobaric hypoxia and with concurrent cognitive task in young healthy males*. Doneddu A, Roberto S, Guicciardi M, Pazzona R, Manca A, Monni A, Fanni M, Leban B, Ghiani G, Spranger M, Mulliri G, Crisafulli A. *Appl Physiol Nutr Metab*. 2024 Aug 1. doi: 10.1139/apnm-2023-0629.
2. *Executive Functions and Mood States in Athletes Performing Exercise Under Hypoxia*. Guicciardi M, Pazzona R, Manca A, Monni A, Scalas LF, Perra F, Leban B, Roberto S, Mulliri G, Ghiani G, Doneddu A, Crisafulli A. *Front Psychol*. 2022 May 27;13:906336. doi: 10.3389/fpsyg.2022.906336..
3. *Acute Exercise with Moderate Hypoxia Reduces Arterial Oxygen Saturation and Cerebral Oxygenation without Affecting Hemodynamics in Physically Active Males*. Mulliri G, Magnani S, Roberto S, Ghiani G, Sechi F, Fanni M, Marini E, Stagi S, Lai Y, Rinaldi A, Isola R, Vargiu R, Spranger MD, Crisafulli A. *Int J Environ Res Public Health*. 2022 Apr 10;19(8):4558. doi: 10.3390/ijerph19084558
4. *Systolic and Diastolic Functions After a Brief Acute Bout of Mild Exercise in Normobaric Hypoxia*. Magnani S, Mulliri G, Roberto S, Sechi F, Ghiani G, Sainas G, Nughedu G, Vargiu R, Bassareo PP, Crisafulli A. *Front Physiol*. 2021 Apr 23;12:650696. doi: 10.3389/fphys.2021.650696.
5. *A brief bout of exercise in hypoxia reduces ventricular filling rate and stroke volume response during muscle metaboreflex activation*. Mulliri G, Magnani S, Roberto S, Sechi F, Ghiani G, Sainas G, Nughedu G, Hosseini Kakhak SA, Bassareo PP, Crisafulli A. Eur *J Appl Physiol*. 2020 Sep;120(9):2115-2126. doi: 10.1007/s00421-020-04435-0.

2- Table 1. Please provide the following values: SV, HR, CO, MAP, SVR that were measured, as revealed in the 'Hemodynamic assessment' section. Or remove the section if not relevant to the results presented.

Answer: The SV, HR, CO, MAP, and SVR values were present in the supporting information of the submitted version of the manuscript. Following the reviewer's suggestion, we have now included these values in the revised manuscript in a dedicated new table (Table 2). The corresponding figure from the supporting material has been removed.

3- When, on which day, was the screening CPET performed? The same or other day as experimental trials?

Answer: The information regarding the day of the CPET test was provided in the original version of the manuscript (*Study design* section in Materials and methods). The CPET was conducted 4 to 7 days before the experimental trials.

4-Was lactate or ventilatory threshold determined during the screening CPET? Please provide parameters in Table 1 if available. Probably the participants did not reach the threshold in normoxia. Interesting, was the lactate/ventilatory threshold achieved or exceeded in hypoxia?

Answer: During the screening CPET, the ventilatory threshold (VT1) was determined in normoxia. We have now included the parameters related to VT1 in Table 1, as requested. We do not have direct measurements of the ventilatory or lactate thresholds under hypoxic conditions. Gas exchange kinetics data could not be obtained during the experimental trial because the hypoxia was administered using a specialized mask, which precluded the use of additional equipment for measuring ventilation, oxygen uptake, and carbon dioxide production. Given that the mean wattage at VT1 was 54% of the maximum wattage achieved during peak exercise, it is likely that the hypoxic trial was conducted below VT1. Nonetheless, this assumption remains speculative without direct hypoxic measurements, a limitation that we acknowledged in the manuscript.

5- I think, the values of basic cardiorespiratory parameters and lactate for normoxia and hypoxia should be provided and compared (HR, VO2, VE, etc.) in a separate table for general characteristic. Could you also provide peak lactate values for normoxia and hypoxia?

Answer: Thank you for your feedback. We have previously addressed the issue regarding the presentation of basic cardiorespiratory parameters and lactate values for hypoxia. Due to the constraints of our experimental setup, i.e. the use of a dedicated mask for administering hypoxia that precluded the use of additional equipment for measuring gas exchange kinetics and lactate, these data were not collected under hypoxic conditions. As such, we are unable to provide a direct comparison of these parameters between normoxia and hypoxia. This limitation has been noted in our manuscript. We appreciate your understanding of this constraint.

6- Line 162–3 This was recovery between the normoxia and hypoxia tests? Unclear.

Answer: We thank the reviewer's feedback concerning the ambiguity in this sentence. The 6-minute recovery period followed the 3-minute pedaling session and marked the conclusion of the

test. Following this, urine and plasma samples were collected within 5 minutes. We have clarified this point in the revised manuscript (*Experimental Trials* section in Materials and Methods).

7- After the experimental trials, what was collected first? Blood or urine? Could the time from trial termination to sampling affect the results?

Answer: Blood was collected first because certain metabolites may have immediate or short-term fluctuations following exercise. Thus, collecting blood samples first can help capture these acute changes more accurately. Urine was collected afterward as it reflects more integrated changes over a somewhat longer period. Additionally, since the bladder can store urine over time, it is less subject to immediate variability compared to blood.

8-Univariate analysis (2-way ANOVA). Please always provide effect size (eta-squared) in the main text and supplementary material, apart from p-value (that itself does not inform of the strength if the effect). Also, please first inform about the main effects (condition, pre-post and their interaction) before providing post hocs.

Answer: We fully agree with the reviewer's comment and have made the suggested revisions. As recommended, partial η^2 values have now been included and discussed throughout the manuscript. Originally, we used GraphPad for ANOVA, which only provided η^2. To obtain partial η^2 values, we reanalyzed the data using JAMOVI. The trends and conclusions from the JAMOVI analysis largely align with those obtained from GraphPad for most metabolites. However, slight variations in p-values were observed between the two software packages, though these did not affect the overall significance of the results. Only in three cases did the outcomes differ between JAMOVI and GraphPad.

Firstly, the ANOVA analysis in JAMOVI revealed significant differences in urine for 3-hydroxyisovalerate (3-HIV) and taurine after the HYPO test. These new findings are consistent with our original interpretation of data: 3-HIV is a product of leucine degradation (similar to 3-HIB for valine), and taurine is a metabolite known to be sensitive to physical activity and hypoxia. Secondly, JAMOVI's post-hoc Bonferroni test no longer finds urine hippurate to be significant.

We believe that the discrepancies between the two software packages arise from differences in their underlying algorithms. In particular, JAMOVI offers a clearer and more transparent ANOVA analysis compared to GraphPad. For instance, while GraphPad provides a single residual value for the entire analysis, pooling all sources of unexplained variance, JAMOVI separates residuals for each factor and their interactions, giving a clearer picture of how each factor contributes to the overall variance in the data.

Given JAMOVI's more transparent analysis and comprehensive output, we chose to adopt it for our data analysis. As a result, the findings related to 3-HIV and taurine have been incorporated and discussed in the revised manuscript, while the discussion on hippurate (which had been

previously identified for further investigation) has been removed. Additionally, the identification of new correlations of 3-HIV with other metabolites led us to represent the final correlation map between plasma and urine metabolites as a heat map, providing a clearer visualization compared to the chord plots used in the original version.

The updated results of ANOVA analysis are presented in Tables S1 and S2 of the revised manuscript. This reanalysis also allowed us to identify and correct some typographical errors from the original version, such as mistakenly reporting p-values calculated for non-log-transformed data rather than the transformed data.

9- First of all, please revise the introduction and discussion, taking into account my earlier comments and suggestions. Please also make an adequate selection of literature cited. Please be more considerate and conservative in your interpretations, which must be based on the results obtained in a specific group of people (who are not athletes).

Answer: Thank you for your comments and suggestions. We have carefully reviewed the introduction and discussion sections of the manuscript in light of your feedback. We have revised these sections to ensure that our interpretations are more conservative and directly aligned with the results obtained from our specific study group. Additionally, we have updated the literature cited to better reflect the scope and focus of our study.

Reviewer #2: Prof. Tomasz Gabrys

Congrats on a very well organized and methodically prepared study. Correctly selected statistical methods, sczegosely described research results. While you can not have comments on the methodological side, in the very purpose and interpretation of the study in my opinion the authors made a mistake. An error that affects the discussion, which is written correctly. Unfortunately, it does not fully correspond to the introduction and assumptions of the study. Reading the work, you get the impression that the authors are introducing the problem of altitude hypoxia. Hypoxic stimulus is then associated with changes in oxygen partial pressure. In the paper we have an experiment based on normobaric hypoxia. In both cases of application of hypoxia, the hypoxic effect is different. While in the first case we can expect an increase in the aerobic component of the effort, in the second case it is not surprising to see an increase in the anaerobic component. Very important is also the susceptibility of the athlete's body to the application of a hypoxic stimulus. In sports training, one must be prepared for hypoxic exposure. The VO2max values of the subjects are so low that they are rather indicative of individuals with a serious deficit in aerobic capacity. The state of preparation in this respect of the study group should be more specifically considered in the discussion. In the reviewer's opinion, the paper needs to be supplemented/revised with an introduction to the mechanisms of normobaric hypoxia. It is also necessary to introduce into the discussion elements specific to the effects of using normobaric hypoxia.

Answer: We thank Prof. Gabrys for his positive feedback on our study's organization, methodology, and statistical analysis. We appreciate his thorough review and the valuable insights he has provided. We acknowledge the distinction made between altitude hypoxia and normobaric hypoxia. Accordingly, we have revised the introduction of the manuscript to clearly define normobaric hypoxia and its differences from hypobaric hypoxia. We have also adjusted the discussion to focus more on the physiological adaptation of a non-athletic population to hypoxia.
