# Supplementary material for: Metabolic response to an acute bout of mild dynamic exercise performed under normobaric moderate hypoxia: A NMR-based metabolomics study
Source: PLoS One. 2025 Jul 1;20(7):e0325447. doi: 10.1371/journal.pone.0325447 (PMC12212504; doi:10.1371/journal.pone.0325447)
Supplement: Attachment — Submitted filename: Response_to_Reviewers_auresp_2.pdf [file pone.0325447.s007.pdf]

Manuscript Number: PLONE-D-24-21080R1
Title: Metabolic response to an acute bout of mild dynamic exercise performed under normobaric moderate hypoxia: a NMR-based metabolomics study

Answers to reviewer comments

Reviewer #1

Authors claim this is the first study to use metabolomics on both urine and blood plasma to assess the combined effects of hypoxia and exercise in non-athletes. Thirteen healthy young adult participants performed identical sessions of mild exercise (3 minutes at 30% Wmax) under both normal oxygen (normoxia) and normobaric hypoxia (simulated altitude) conditions. Blood and urine samples were collected before and after exercise, and analyzed using 500 MHz NMR spectroscopy. Results showed significant metabolic response to mild exercise under hypoxia, even at low workloads, highlighting the potential of hypoxic exercise for non-athletes. Plasma analysis revealed decreased branched-chain amino acids, phenylalanine, and tyrosine, alongside increased lactate, succinate, citrate, and 3-hydroxybutyrate. Urine analysis showed increased 3-hydroxyisobutyrate, 3-hydroxyisovalerate, alanine, lactate, succinate, acetone, taurine, and glycine, with decreased dimethylamine. These changes suggest alterations in energy metabolism pathways and potential metabolic stress.

Overall, the study emphasizes the potential of metabolomics for understanding metabolic adaptations of non-athletic individuals to hypoxic conditions. This is crucial for developing effective rehabilitation and health strategies to improve recovery and optimize health outcomes in non-athletic populations. The revised manuscript is well written and I recommend the publication of the article with a few minor suggestions and corrections to further enhance the manuscript's quality

Specific comments:

✓ **Comment**: Authors used CHENOMX for assignment purpose, however, it can also be used for concentration profiling. Specifically, the circulatory levels of discriminatory metabolic features can be profiled using CHNEOMX considering format as an internal reference for blood plasma samples and creatinine for urine samples. The results corresponding to statistical comparison can be discussed in the main manuscript and Figures can be included in the supplementary materials for future reference.

Answer: We appreciate the reviewer's suggestion regarding the use of CHENOMX for metabolite quantification. However, as reported in the manuscript (page 10), we only had access to an evaluation version of the software which has significant limitations, including the inability to save files and a restriction to quantifying a maximum of five metabolites at a time. These constraints made it impractical to perform a systematic and comprehensive quantification across all samples. For this reason, we primarily used CHENOMX to support the spectral assignment of NMR signals. Since our primary focus was on the comparative characterization of metabolite variations across experimental conditions, we focused our analysis on relative variations in NMR signal intensities rather than absolute quantification. Nonetheless, we acknowledge the value of quantitative metabolite profiling and consider it an important aspect for future studies.

✓ **Comment**: The statistical correlation analysis between marker metabolic features with key clinical parameters need to be performed, if possible. The statistically significant correlation will establish the biological relevance of this study. The correlation analysis needs to performed separately for normoxia and hypoxia groups, accordingly there will be two correlation heat maps for plasma samples and two correlation heat maps for urine samples. The significance of the statistical correlation is expected to be high for discriminatory metabolic features in Hypoxia group. Authors may refer to this paper for correlation analysis: BioCPR–A Tool for Correlation Plots (https://www.mdpi.com/2306-5729/6/9/97)

Answer: We thank the reviewer for the suggestion to explore correlations between metabolite levels and hemodynamic parameters. We explored the online application of BIOCPR (biocpr.shinyapps.io/corplot) and carefully reviewed the associated publication. However, we encountered difficulties in uploading and processing our dataset within the platform's interface, which unfortunately limited our ability to use the tool effectively. As an alternative, and in line with the reviewer's recommendation, we proceeded to perform detailed correlation analyses using JAMOVI. Specifically, we conducted separate Sperman correlation analyses for plasma and urine samples, considering both the NORMO and HYPO exercise sessions, resulting in four independent analyses.

Since the most marked changes in hemodynamic parameters occurred during the third minute of exercise and returned rapidly to baseline during the recovery phase, we reasoned that this time point represented the most relevant window to capture the physiological impact of the exercise. Given the longitudinal nature of the study and the overall homogeneity of the cohort, we included both pre- and post-exercise metabolite levels alongside hemodynamic measurements taken pre-exercise and at minute three of exercise (EXE3). This strategy allowed us to capture intra-subject adaptations and better assess whether metabolic shifts were associated with concurrent cardiovascular responses. The results are presented as correlation heatmaps in Figure 7. No statistically significant associations were detected in the NORMO condition, either in plasma or urine, consistent with the modest physiological impact of the normoxic protocol. Differently, numerous significant correlations emerged following the HYPO test, underscoring the biological relevance of the metabolomic alterations observed under hypoxia and highlighting a close interplay between metabolic and cardiovascular responses during this condition. All relevant results have been added to the revised *Results* section and further elaborated in the *Discussion*.

✓ **Comment**: For future validation studies and establish the reproducibility of the method, authors should submit all the NMR raw data to a public repository like metabolights of workbench or ZENODO database. The corresponding details should be incorporated into the manuscript as a data availability statement.

Answer: We thank the reviewer for the valuable suggestion. In response, we have deposited the NMR data in the ZENODO public repository. Furthermore, given the inclusion of new correlation analyses in the revised manuscript, we have also uploaded the hemodynamic data collected from study participants. Datasets are currently accessible via a DOI and a private URL, which have been included in the manuscript's *Data Availability Statement.*

Reviewer #2

Dear Authors,

I had the opportunity to review the revised version of your manuscript, which investigates the effect of a brief exercise bout in normobaric hypoxia on metabolic and physiological variables in physically active young adults. The relevance of this topic is notable, given the growing interest in hypoxia strategies to optimize health and performance. However, I would like to respectfully suggest some areas for improvement. Specifically, the description of the study design could be clarified, and it would be beneficial to define the type of investigation (whether experimental and/or a clinical trial). Additionally, the organization of the Materials and Methods section could be streamlined. To address these points, a comprehensive evaluation of the submitted manuscript is warranted. This evaluation encompasses methodological robustness, data presentation, statistical analyses, and clarity of reporting:

✓ **Comment**: The study design, which centers on the impact of brief exercise under moderate normobaric hypoxia, is conceptually relevant. The collection of metabolic and physiological measures, along with the statistical comparisons demonstrating consistent differences between normoxic and hypoxic conditions, is noteworthy. However, your manuscript would benefit from a clearer classification of its design, such as whether it is best described as an experimental crossover trial or another format. While the data presented support the general conclusions, the findings would be more robust if you explicitly detailed the randomization and enrollment procedures, the rationale for sample size, and the assumptions underlying the statistical tests performed.

Answer: We appreciate the reviewer's valuable suggestion, which allows us to clarify our methodology more effectively. We have now explicitly stated at the beginning of the "Study Design" that this is a crossover randomised controlled study, with each subject serving as their own control. Additionally, we have refined the methodological details by providing a more comprehensive description of: 1) the randomization method for the sequence of conditions (normoxia vs. hypoxia); 2) the enrolment procedure; 3) the sample size calculation, based on a statistical power analysis. Furthermore, we have revised the inclusion and exclusion criteria to enhance clarity. These modifications have been incorporated into the "Study Design" (page 5) and the "Participants" (page 6) sections. For further details, please refer to our response to Comment B.

✓ **Comment**: Additionally, strengthening the link between specific metabolite changes and their physiological significance would enhance the discussion. The consistency between plasma and urine findings is intriguing; however, your manuscript should establish how these results connect with broader physiopathological or performance-related frameworks.

Answer: We thank the reviewer for this insightful comment, which has helped us strengthen the physiological interpretation of our findings. In response, we have revised the *Results*, *Discussion* and *Conclusion* sections to better integrate the observed metabolite changes with their physiological significance and potential implications within broader performance-related and pathophysiological frameworks. Specifically, following the recommendation of Reviewer #1, we incorporated the results of

correlation analyses between key discriminatory metabolites and hemodynamic parameters measured during exercise. These analyses revealed significant associations in the HYPO condition, particularly involving amino acids, tricarboxylic acid (TCA) cycle intermediates, and ketone bodies, whereas no relevant correlations emerged under normoxia. These findings indicate that the metabolic shifts observed following hypoxic exercise are not only statistically robust but also biologically meaningful, reflecting coordinated systemic adaptations to the cardiovascular challenge. We interpret this as evidence that the metabolome may act as a sensitive integrator of physiological stress, capable of capturing subtle but functionally important responses that may not be fully reflected by standard hemodynamic metrics.

We have also expanded on the potential translational value of our findings, noting that several metabolites altered in response to hypoxia are known to be dysregulated in cardiometabolic diseases. While our study involved healthy, moderately active individuals, this overlap suggests that the metabolic responses we observed may reflect conserved adaptive mechanisms, which could become dysfunctional in clinical settings. These additions to the manuscript enhance the physiological relevance of our findings and support the potential for future research to explore hypoxia-based protocols aimed at both optimizing performance and supporting recovery or metabolic health in rehabilitation contexts.

✓ **Comment**: Your statistical approach is generally appropriate for investigating acute exercise effects. However, the manuscript would benefit from greater clarity regarding normality tests (e.g., Shapiro–Wilk), corrections for multiple comparisons (if multiple metabolites were tested simultaneously), and effect size metrics. While your results indicate significant changes, you should confirm that the sample size is sufficient to detect the observed effect sizes with adequate power. Additionally, explicitly stating how missing data or potential outliers were handled would reinforce the transparency of your study.

Answer: We appreciate the reviewer's valuable feedback on the statistical approach. In the previously submitted manuscript, we reported the following information: 1) the normality of the data was assessed using the Shapiro–Wilk test; 2) where necessary, log transformation was applied to meet normality assumptions; 3) Bonferroni correction was used to correct for multiple comparisons; 4) partial eta squared (η^2) for ANOVA tests was reported in Tables S2-S3 for interpretation of the effect magnitudes. To improve clarity, we have revised the statistical methods section accordingly (page 11).

Regarding sample size adequacy, we conducted a post-hoc power analysis, based on the η^2 values derived from the ANOVA tests for the most significantly altered metabolites. This analysis confirmed that our study had adequate statistical power (≥80%) to detect the main effects of exercise and the exercise × oxygen interaction on plasma metabolites. For the main effect of oxygen exposure alone, the power was slightly lower (75%), suggesting a moderate probability of a Type II error. In contrast, the analysis for urine metabolites yielded excellent power values (>90%) for all three effects (exercise, oxygen, and their interaction), supporting the robustness of those findings. These results have been reported and discussed in the new section of the revised manuscript titled *Study strengths and limitations* (page 26).

Finally, we confirm that there were no missing data and no statistical outliers in the dataset. All values were carefully checked, and no data points exceeded the standard thresholds typically used to define outliers (e.g., >3 SD from the mean). This information has also been included in the revised *Methods* section for completeness.

✓ **Comment**: From a linguistic and structural standpoint, your manuscript is generally well-written and understandable. The English is clear enough for an international audience, though certain sections could be condensed or reorganized for improved readability. Specifically, the Materials and Methods section would benefit from a more conventional structure, beginning with the study design and participant flow, followed by details on the exercise protocol, biological sampling, and analytical methods. Improving the resolution of Figure 1 and ensuring that the legend is sufficiently explanatory would help readers better understand the study's timeline and interventions.

Answer: We thank the reviewer for the constructive feedback regarding the organization of the *Materials and Methods* section and the clarity of Figure 1. In response to this suggestion, we have restructured the *Materials and Methods* to follow a more conventional format. Furthermore, the paragraph listing chemical reagents has been removed. Instead, details about each chemical have been incorporated directly into the relevant methodological sections where they are used. These adjustments aim to enhance clarity and readability. We have also replaced Figure 1 with a new high-resolution version, and revised both the figure and its legend to provide a clearer and more detailed explanation of the study timeline, participant allocation, and experimental procedures. Additional details regarding these modifications are provided in our responses to the following comments.

Below, I provide a detailed assessment of the revised manuscript, taking into account the PLOS One Guidelines for Reviewers:

✓ **Comment**: A) *General Comments and Context* - The manuscript follows the IMRaD structure; however, the presentation of the study design and its classification is not fully aligned with best practices for methodological description. This is particularly relevant in distinguishing between observational studies and clinical trials (or comparative experimental studies). In my opinion, this is a comparative experimental study, as it involves a planned intervention applied to a group of participants, with measurements of physiological and metabolic outcomes. However, the manuscript does not clearly state, at the beginning of the Materials and Methods section, the exact type of study being conducted. I recommend reorganizing the Study Design subtopic as the first item in Materials and Methods, clarifying the type of study.

Answer: We appreciate the reviewer's insightful feedback regarding the methodological description. Following the recommendation, we have reorganized the *Materials and Methods* section, placing the *Study Design* as the first subheading to improve clarity and alignment with best practices. Additionally, we have explicitly clarified that this study is a crossover randomized controlled trial, as participants completed two experimental conditions in a randomized order. This distinction has been incorporated at the beginning of the *Study Design* section (page 5).

✓ **Comment**: B) *Possible Need to Follow CONSORT*. Based on the study elements presented, there are indications that this could be classified as a clinical trial (even if a pilot or small-scale study). If so, adherence to the Consolidated Standards of Reporting Trials (CONSORT) is recommended to standardize the reporting of randomized clinical trials. If this study is not strictly a clinical trial but still a controlled experimental study, the text should, at the very least, align with CONSORT's principles of

clarity and transparency, clearly describing allocation procedures, sequence randomization, and potential losses.
Additionally, if session order (e.g., normoxic vs. hypoxic) was randomized and an intervention was applied, it is necessary to partially adhere to the CONSORT checklist. I recommend verifying whether CONSORT compliance is required for this study and, if not, adopting at least its key aspects.

Answer: We appreciate the reviewer's suggestion regarding adherence to CONSORT guidelines. Our study does not qualify as a clinical trial, as it does not involve therapeutic interventions, patient treatments, or health-related outcomes. Instead, it is a randomized controlled experimental study designed to investigate physiological and metabolic responses to exercise under different oxygen conditions.

We acknowledge the importance of clarity and transparency in methodological reporting. Following the reviewer's recommendation, we have restructured the description of the *Study design* (page 5) and *Participants* (page 6) sections, addressing key CONSORT principles related to the methodological framework. To enhance clarity, we have ensured that the manuscript explicitly describes the following aspects: randomization procedure, participant allocation and flow (this information has also been incorporated into Figure 1 to enhance study transparency), detailed methodological description. We believe that these improvements enhance the rigor and transparency of our study, ensuring that the methodology is clearly defined and reproducible, in line with best reporting practices.

✓ **Comment** *C) Figure Quality*. The figures, especially Figure 1, remain in low resolution even after accessing the linked version. Furthermore, it seems that the diagram aims to illustrate the study chronology or protocol. However, if it serves as a clinical trial flowchart, it should include details such as the number of participants screened, any exclusions, and the distribution between conditions. I recommend providing figures in high resolution and, if possible, making the legend more detailed, specifying each phase of the study and the number of participants in each stage.

Answer We thank the reviewer for the feedback regarding the quality and clarity of Figure 1. In response to this comment, we have replaced the previous version with a new higher-resolution figure to ensure better readability. Additionally, we have made the following adjustments:

- explicitly included the number of participants at each stage of the study and the number of post hoc excluded subjects;
- revised the figure legend to explicitly state that it provides an overview of the experimental design. The figure illustrates the study timeline, participant allocation, and randomization process, rather than serving as a formal trial flowchart.

✓ **Comment** D) Table Presentation. Table 1 is currently formatted descriptively but does not highlight the comparative aspect between groups or conditions. Given that the study aims to compare physiological/anthropometric parameters under different conditions, Table 1 could be reorganized to present these comparisons side by side, with the type of statistical analysis used clearly indicated. I recommend revising Table 1 to align it more effectively with the standards of an experimental study.

Answer: We thank the review for the feedback regarding Table 1. This table was intended solely to offer a descriptive overview of the participants' baseline anthropometric and cardiopulmonary characteristics, measured prior to the experimental sessions. These values reflect each participant's general physiological

profile and were not expected to be affected by the short-term, acute exercise interventions employed in the study. Moreover, since the normoxic and hypoxic sessions were conducted 7 to 10 days apart, we assumed that no meaningful variation in these baseline characteristics would occur between sessions. For these reasons, we did not consider statistical comparisons between conditions to be methodologically relevant or meaningful for this section of the dataset. Table 2 already provides a detailed comparison of physiological parameters measured under both NORMO and HYPO conditions, at four time points. Statistical differences within conditions (e.g., vs rest) are clearly indicated, and comparisons between conditions are described in the text where appropriate. This presentation aligns with the methodological aims of the study and the within-subject crossover design.

✓ **Comment**: I believe that partially or fully adopting CONSORT guidelines or another checklist for reporting experimental studies would greatly improve transparency and replicability. Additionally, I strongly recommend providing greater detail on randomization, blinding, washout periods, participant losses, and sample size calculations, reinforcing these aspects in the corresponding sections of the text. Finally, I would like to emphasize that the topic of this article is highly relevant and presents interesting findings, particularly in the context of applications for non-athletic populations, rehabilitation, and comparative physiology studies.

Answer: We are sincerely grateful to the reviewer for the insightful and constructive feedback provided throughout the revision process. In response to the his/her comment, we have carefully revised the manuscript to improve its clarity, transparency, and reproducibility. We also greatly appreciate the reviewer's recognition of the study's relevance and the potential implications of our findings for non-athletic populations, rehabilitation, and comparative physiology. We hope that the revisions made have fully addressed the reviewer's comments and contributed meaningfully to the overall quality and clarity of the manuscript.
